# Knowledge, Awareness, and Perception of Genetic Testing for Hereditary Disorders Among Malaysians in Klang Valley

**DOI:** 10.3389/fgene.2020.512582

**Published:** 2020-12-03

**Authors:** Jia-Jia Chin, Hong-Wai Tham

**Affiliations:** Biopharmaceutical Research Unit, Biology Research Laboratory, Faculty of Pharmacy, SEGi University, Petaling Jaya, Malaysia

**Keywords:** knowledge, awareness, perception, public survey, genetic testing, hereditary disorders

## Abstract

Genetic testing aids patients in making important decisions in the prevention, treatment, or early detection of hereditary disorders. Low awareness of the importance of genetic testing contributes to the increase in the incidence of hereditary disorders. This study aims to explore the knowledge, awareness, and perception of genetic testing for hereditary disorders among local residents of the Klang Valley, Malaysia, and the potential variables that influence their understanding of genetic testing. A survey was conducted in different municipalities of the Klang Valley through self-administered questionnaire assessing the public's knowledge, awareness, and perception of genetic testing. Overall, the results revealed adequate knowledge and positive awareness of genetic testing, in which both were influenced by the respondent's educational level (*P* < 0.001), field of study (*P* < 0.001), and status of heard or unheard of genetic testing (*P* < 0.001). The perception of genetic testing was generally positive and influenced by the respondent's differences in age (*P* < 0.016), educational level (*P* < 0.001), field of study (*P* < 0.001), and status of heard or unheard of genetic testing (*P* < 0.001). Although positive responses were obtained, ~20.2% of the respondents had never heard of genetic testing. Of the respondents, 24.5% were unwilling to undergo genetic testing, with 25.1% believing that genetic testing tampers with nature and 18% believing that it opposes religion and their beliefs. Such attitude calls for the need to conduct programs to eliminate any misconception, as well as to educate the public to lessen any perceived misunderstanding of the concepts of genetic testing.

## Introduction

Genetic disorder is a health condition that is inherited by a person, usually caused by mutations in the deoxyribonucleic acid (DNA) or changes in the number or overall structure of chromosomes (Odelola et al., 2013). Several types of commonly known diseases have been determined to be related to hereditary gene mutations. In developed or developing countries including Malaysia, the major well-known non-communicable health concerns include hypertension, diabetes, and hypercholesterolemia (Lim et al., 2004; Hussein et al., 2015; Naing et al., 2016; Tee and Yap, 2017; Rifin et al., 2018). On the other hand, ~5–10% of cancers are known to contain hereditary components (Lu et al., 2014; Stanislaw et al., 2016). These include breast cancer (Miki et al., 1994; Edwards et al., 2003; McPherson, 2006) and colorectal cancer (Hampel, 2009; Jasperson et al., 2010; Stanislaw et al., 2016). Blood-related disorders, such as sickle cell disease, thalassemia, and hemophilia, are also hereditary diseases. These diseases are acquired by inheriting abnormal genes, such as hemoglobin C gene.

Previous studies reported a deficiency of knowledge of genetic testing among the public, including populations in developed and developing nations (Vermeulen et al., 2014; Agurs-Collins et al., 2015; Hann et al., 2017; Eum et al., 2018; Altaany et al., 2019). Meanwhile, similar studies targeting prenatal or neonatal genetic testing revealed that health education, facilities, and infrastructures should be further improved to enhance the adaptation of non-invasive genetic testing among the public (Kusyk et al., 2013; Abdo et al., 2018). Moreover, Colotto et al. (2015) reported a good level of awareness of and interest in genetic testing among medical students (Colotto et al., 2015). Despite the deficiency of knowledge of genetics among practitioners, gynecologists, and pediatricians reported in 2005 (Baars et al., 2005), a recent report proposed that physicians' confidence and interest in genetic testing can be augmented through additional health education (Haga et al., 2019).

A recent study reported the need of Malaysia to strengthen its role in the field of genetic test (Balasopoulou et al., 2017). Continuous efforts have been taken by researchers/stakeholders in Malaysia by introducing medical genetic services nearly a decade ago (Lee and Thong, 2013). Since then, genetic services have improved with the availability of genetic counseling, testing, and diagnosis. The recognition of Clinical Genetics as a subspecialty and increased funding for genetics services also contributed to the growth of genetic testing in Malaysia. In addition, a number of survey studies were conducted to examine the level of knowledge, perception, or awareness of patients or young Malaysians (Al-Naggar and Osman, 2013; Mustapa et al., 2019; Qian et al., 2019) or physicians (Amini et al., 2015) regarding genetic risk of inheritable disorders in Malaysia. Meanwhile, similar surveys were reported to conclude the views of undergraduates on genetic testing (Sim and Ting, 2018; Sulaiman and Zainuddin, 2018). However, the view of public toward genetic testing is still limited in Malaysia. In 2011, Wong et al. addressed public attitudes and perceptions on thalassemia (Wong et al., 2011). In 2013, Lee and Thong reported a limited public awareness in the same region (Lee and Thong, 2013). Therefore, an update on the public general knowledge, awareness, and perception toward genetic testing of hereditary disorders is necessary. To fulfill the research gap, this survey study aimed to examine the knowledge, awareness, and perception toward genetic testing of hereditary disorders in the public of Klang Valley, Malaysia.

## Methodology

### Study Design and Study Population

This is a cross-sectional study conducted between September 2018 and November 2018, using convenient sampling targeting the local residents of the Klang Valley, including districts, such as Shah Alam, Subang Jaya, Ampang, Putrajaya, Kajang, Petaling, Cheras, and Kuala Lumpur. In accordance to the Department of Statistics of Malaysia, the total population of Klang Valley in 2018 was measured at 7.2 million. The sample size was calculated using Raosoft sample size calculator (Raosoft, Inc. 2004, http://www.raosoft.com/samplesize.html), providing a confidence level of 95%, with a margin of error of 5% (Albassam et al., 2018; Mason et al., 2018), which indicated the need to approach 384 responses in this study. To take into account of any redundancy, a total of 450 responses were collected. Responses were received from various districts of Klang Valley. Questionnaires were randomly distributed to the public in supermarkets (wet and dry markets), transport hubs (connecting train or bus stations), and areas with eateries, during both peak and off-peak hours. Questionnaires were collected on the spot after responses were filled in by respondents. Respondents who participated in this study were within the age range of 18–50 years old, Malaysian, and from different fields of profession.

### Questionnaire Design

The questionnaire was adapted from a few reliable and valid research papers related to this study (Henneman et al., 2013; Chokoshvili et al., 2017; Sim and Ting, 2018). The self-administered questionnaire comprised four sections with a total of 42 closed-ended questions. The first section with a total of eight questions focused on the details of the respondents, including demographic characteristics. The second section with a total of 10 questions in trichotomous form, i.e., yes/no/maybe, focused on the knowledge of genetic testing. The third (11 questions) and fourth (13 questions) sections focused on the awareness and perception of genetic testing, respectively, which was assessed using a five-point Likert scale ranging from 1 to 5, where 1 represents “strongly disagree” and 5 represents “strongly agree.” Prior to the survey, questionnaires were distributed to 50 respondents (11% of total respondents), with comments received to rephrase specialized healthcare terminologies and reconstruct some questions in the questionnaire. Responses received in the pilot study were not included in the final statistical analyses.

### Statistical Analysis

The collected data were analyzed using the Statistical Package for Social Sciences (SPSS) version 22.0 software. Descriptive data analysis, Cronbach's alpha test, Mann–Whitney *U* test, and Kruskal–Wallis test were used to determine all of the related factors associated with knowledge, awareness, and perception.

A scoring system was used to analyze the data, which were coded and inputted into the SPSS software. For questions regarding knowledge of genetic testing, a score of 2 was given for “yes,” a score of 1 was given for “maybe,” and a score of 0 was given for “no.” Then, the total score was summed and classified according to the following categories: inadequate = 0–53%, moderate = 54–66%, and adequate = 67–100% (Haga et al., 2013).

### Ethical Consideration

This study was approved by the SEGi Ethics Committee, with project number SEGi/RIMC/FOP/26/2018. The names, phone numbers, and identity card numbers of participants were excluded to ensure anonymity and confidentiality. Before the survey, informed written consent was obtained from each respondent, and they were informed that the research was voluntary, confidential, and purely for academic purposes.

## Results

### Basic Demographic Data

**Figure 1:**
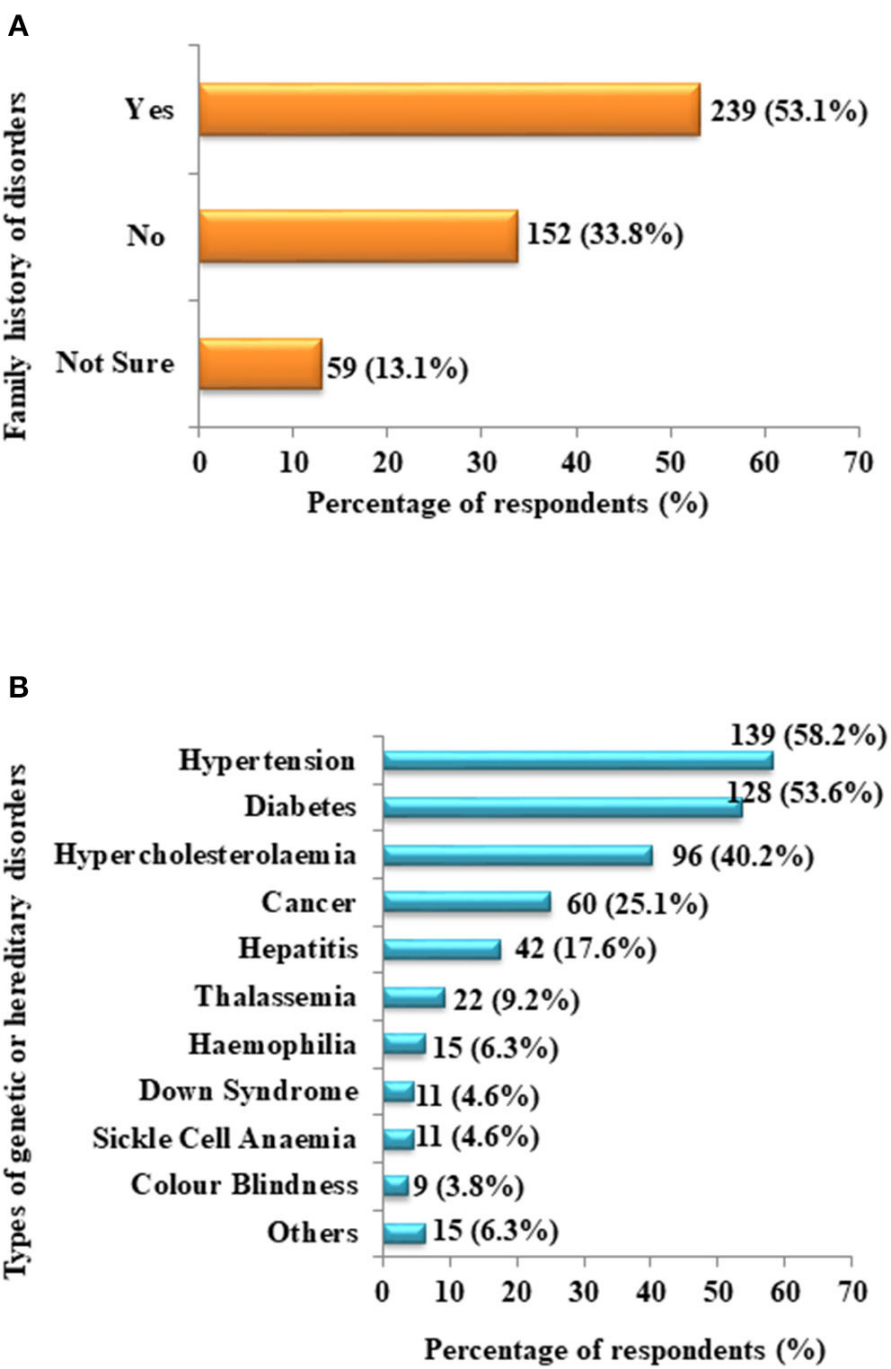
Distribution of respondents (in percentage). **(A)** Respondents with family history of any genetic or hereditary disorder. **(B)** The types of genetic or hereditary disorders declared by the respondents to have occurred in their family history.

Most of the respondents were in the age range of 18–26 years old (47.8%). Of the respondents, 56.2% graduated with a Bachelor's degree. A significant number (37.6%) of respondents were from science-related fields of study. Moreover, of the respondents, 63.6% was single during the duration of this study. In this study, 239 (53.1%) respondents declared having a history of genetic or hereditary disorders in their family, where hypertension (58.2%) was the most prevailing disorder, followed by diabetes (53.6%) and hypercholesterolemia (40.2%) (Figure 1).

### Knowledge of Genetic Testing for Hereditary Disorders

**Figure 2:**
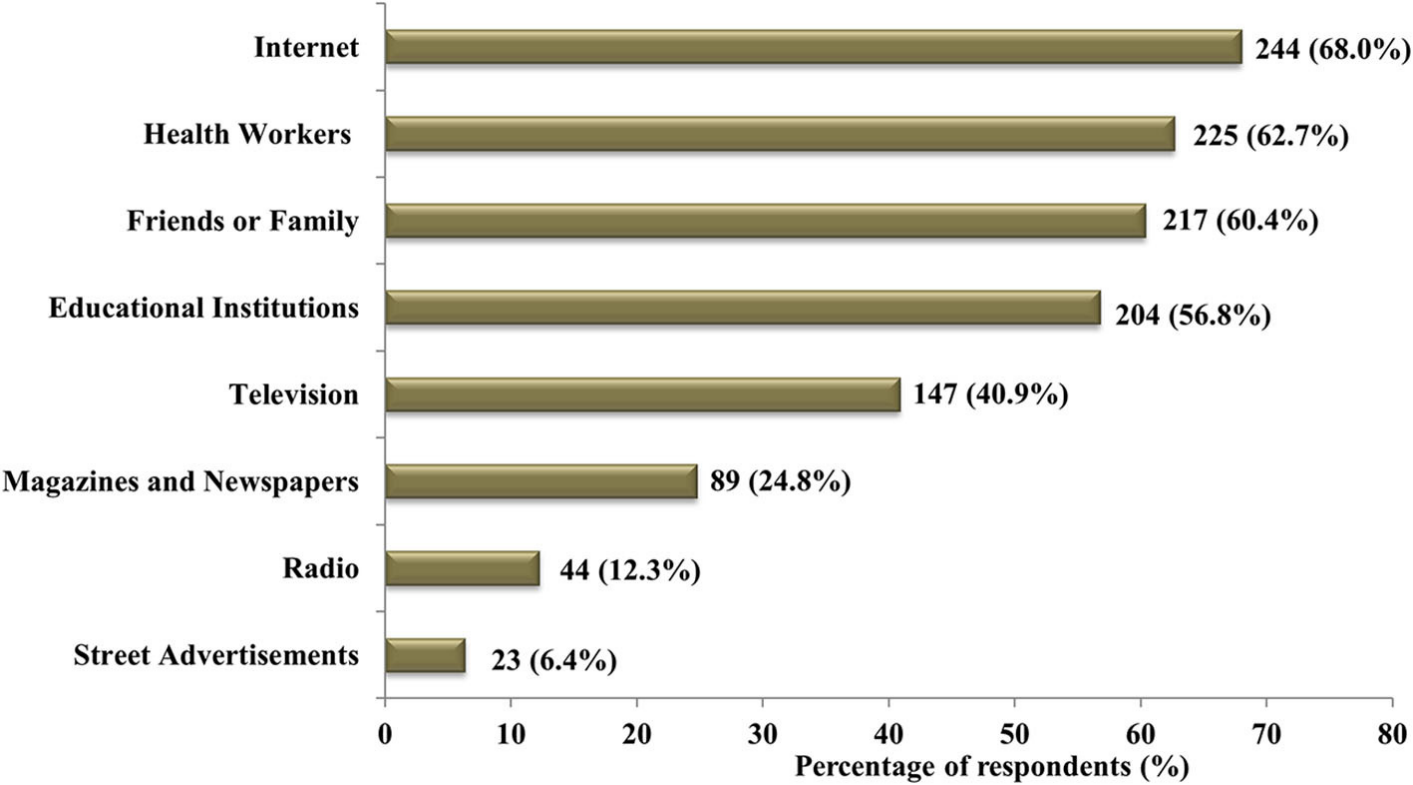
Sources of knowledge regarding genetic testing declared by the respondents.

In this study, 359 (79.8%) respondents have heard of genetic testing, and the Internet was their key source of information (68.0%) (Figure 2). Table 1 shows that most of the respondents (71.1%) knew that genetic testing can be used to diagnose inherited diseases. However, most of the respondents (57.2%) were unsure of the usefulness of genetic testing in reducing the prevalence of genetic diseases. They (58.3%) were also unaware that genetic testing can be used to test for various types of cancer (Table 1).

**Table 1:** Total and percentage of the respondents' answers pertaining to knowledge of genetic testing.

**Statements**	**Total (%)**		
	Yes^*^	**Maybe**	**No**
Genetic testing allows the genetic diagnosis of vulnerabilities to inherited diseases.	320 (71.1)	116 (25.8)	14 (3.1)
Genetic testing can reduce the prevalence of genetic diseases.	192 (42.8)	194 (43.2)	63 (14.0)
Genetic testing can help understand a genetic feature and its sequences.	356 (79.3)	83 (18.5)	10 (2.2)
A person's genetic profile can be used to check whether they are at risk of genetic or hereditary diseases.	356 (79.3)	84 (18.7)	9 (2.0)
Genetic testing can identify specific disease that runs in the family.	360 (80.2)	79 (17.6)	10 (2.2)
Genetic diseases can be passed on in a family.	382 (85.3)	63 (14.0)	3 (0.7)
Prenatal screening is the testing for diseases or conditions of the fetus or embryo before it is born.	265 (59.3)	165 (36.9)	17 (3.8)
Genetic testing can be done during pregnancy to find out whether the baby will develop diseases, such as sickle cell disease, thalassemia, or neural tube defects.	240 (53.5)	162 (36.2)	46 (10.3)
Blood test or DNA analysis is one of the methods used in genetic testing.	382 (85.1)	59 (13.1)	8 (1.8)
Genetic testing can identify various types of cancers, such as colon cancer and breast cancer.	187 (41.7)	200 (44.5)	62 (13.8)

* *Correct answer*.

**Figure 3:**
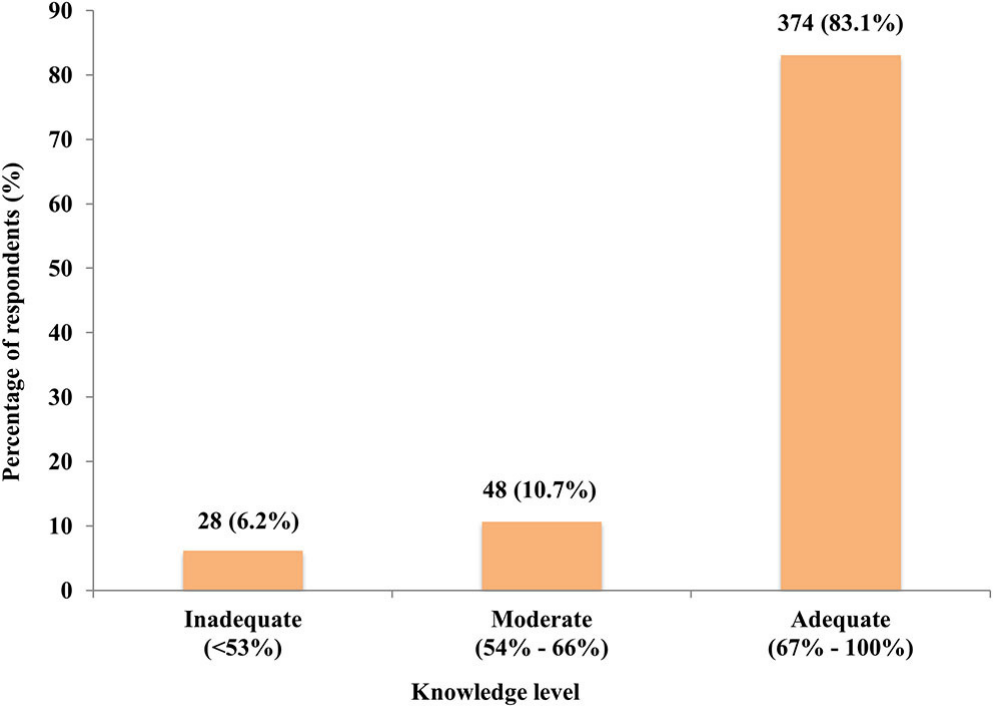
Distribution of the knowledge levels of the respondents.

The majority of the respondents (83.1%) have adequate knowledge of genetic testing (Figure 3). As shown in Table 2, the knowledge of genetic testing can be associated with their educational level, their field of study, and whether they have heard of genetic testing or not, with each having a *P*-value of <0.001 (Table 2).

**Table 2:** *P*-value for the respective variables pertaining to questions on knowledge of genetic testing.

**Variables**		**Knowledge Level**			**Total**	***P*-value**
		**Inadequate (<53%)**	**Moderate (54–66%)**	**Adequate (67–100%)**		
		***N*** **(%)**	***N*** **(%)**	***N*** **(%)**	***N*** **(%)**	
Gender	Female	16 (6.8)	26 (11.1)	193 (82.1)	235 (52.2)	0.548
	Male	12 (5.6)	22 (10.2)	181 (84.2)	215 (47.8)	
Age	18–26	11 (5.1)	24 (11.2)	180 (83.7)	215 (47.8)	0.816
	27–34	4 (4.2)	13 (13.5)	79 (82.3)	96 (21.3)	
	35–42	6 (6.3)	9 (9.5)	80 (84.2)	95 (21.1)	
	43–50	7 (15.9)	2 (4.5)	35 (79.5)	44 (9.8)	
Race	Malay	10 (7.6)	17 (13.0)	104 (79.4)	131 (29.1)	0.576
	Chinese	12 (5.4)	22 (10.0)	187 (84.6)	221 (49.1)	
	Indian	6 (6.7)	8 (9.0)	75 (84.3)	89 (19.8)	
	Others	0 (0)	1 (11.1)	8 (88.9)	9 (2.0)	
Marital status	Single	14 (4.9)	31 (10.8)	241 (84.3)	286 (63.6)	0.547
	Married	14 (8.6)	17 (10.4)	132 (81.0)	163 (36.2)	
	Divorce	0 (0)	0 (0)	1 (100)	1 (0.2)	
Level of education	Others	9 (27.3)	4 (12.1)	20 (60.6)	33 (7.3)	<0.001^*^
	A-levels or equivalent	5 (10.6)	5 (10.6)	37 (78.7)	47 (10.4)	
	Diploma	4 (5.4)	19 (25.7)	51 (68.9)	74 (16.4)	
	Bachelor's	10 (4.0)	20 (7.9)	223 (88.1)	253 (56.2)	
	Master's	0 (0)	0 (0)	29 (100)	29 (6.4)	
	Doctorate	0 (0)	0 (0)	14 (100)	14 (3.1)	
Field of study	Science	4 (2.4)	5 (3.0)	160 (94.7)	169 (37.6)	<0.001^*^
	Arts	7 (4.6)	24 (15.9)	120 (79.5)	151 (33.6)	
	Others	8 (8.3)	14 (14.6)	74 (77.1)	96 (21.3)	
	No formal	9 (26.5)	5 (14.7)	20 (58.8)	34 (7.6)	
Status	Heard of genetic testing	8 (2.2)	25 (7.0)	326 (90.8)	359 (79.8)	<0.001^*^
	Never heard of genetic testing	20 (22.0)	23 (25.3)	48 (52.7)	91 (20.2)	

* *Significant value*.

### Awareness of Genetic Testing for Hereditary Disorders

As shown in Table 3, half (50.2%) of the respondents showed willingness to undergo genetic testing. Questions regarding the potential providers of genetic testing were also asked, in which there is a strong support (64.6%) that genetic testing should be performed only in hospitals with a doctor's prescription and that the sale of genetic testing kits through the Internet and in stores should be banned (55.7 and 54.4%, respectively). Moreover, a significant number of respondents (78.4%) agreed that the public's view and awareness of genetic testing is essential (Table 3).

**Table 3:** Total and percentage of the respondents' answers pertaining to awareness of genetic testing.

**Statements**	**Total (%)**				
	**Strongly disagree**	**Disagree**	**Neutral**	**Agree**	**Strongly agree**
I have sufficient knowledge of genetic testing.	46 (10.2)	122 (27.1)	187 (41.6)	84 (18.7)	11 (2.4)
I am aware that I have a unique genetic feature compared with others.	23 (5.0)	64 (14.3)	196 (43.7)	130 (29.0)	36 (8.0)
I would like to have genetic testing.	21 (4.8)	88 (19.7)	113 (25.3)	173 (38.8)	51 (11.4)
Genetic testing tells me the risk of acquiring certain diseases.	9 (2.0)	24 (5.3)	81 (18.0)	248 (55.3)	87 (19.4)
I am aware that not all genetic disorders can be cured.	13 (2.9)	22 (4.9)	84 (18.8)	212 (47.3)	117 (26.1)
Genetic test should only be performed in the hospital with a doctor's prescription.	14 (3.1)	41 (9.1)	104 (23.2)	190 (42.3)	100 (22.3)
Genetic test can be sold through the Internet.	101 (22.5)	149 (33.2)	141 (31.4)	43 (9.6)	15 (3.3)
Genetic test can be sold in stores.	97 (21.6)	147 (32.8)	126 (28.1)	68 (15.1)	11 (2.4)
Genetic testing is closely related to science and medicine.	9 (2.0)	25 (5.6)	119 (26.6)	216 (48.2)	79 (17.6)
There are technologies in documenting genetic profiles for various genetic disorders.	3 (0.7)	20 (4.4)	99 (22.1)	257 (57.4)	69 (15.4)
Public's view and awareness of genetic testing is important.	3 (0.7)	18 (4.0)	76 (16.9)	212 (47.2)	140 (31.2)

Overall, the respondents showed relatively good awareness of genetic testing for hereditary disorders, which is influenced by their educational level, their field of study, and whether they have heard of genetic testing or not, with each having a *P*-value of <0.001 (Table 4).

**Table 4:** *P*-value for the respective variables pertaining to questions on awareness of genetic testing.

	**Variables**	**Total**	***P*-value**
		***N* (%)**	
Gender	Female	235 (52.2)	0.880
	Male	215 (47.8)	
Age	18–26	215 (47.8)	0.081
	27–34	96 (21.3)	
	35–42	95 (21.1)	
	43–50	44 (9.8)	
Race	Malay	131 (29.1)	0.286
	Chinese	221 (49.1)	
	Indian	89 (19.8)	
	Others	9 (2.0)	
Marital status	Single	286 (63.6)	0.529
	Married	163 (36.2)	
	Divorce	1 (0.2)	
Level of education	A-levels or equivalent	47 (10.4)	<0.001^*^
	Diploma	74 (16.5)	
	Bachelor's	253 (56.3)	
	Master's	29 (6.4)	
	Doctorate	14 (3.1)	
	Others	33 (7.3)	
Field of study	Science	169 (37.6)	<0.001^*^
	Arts	151 (33.6)	
	Others	96 (21.3)	
	No formal	34 (7.5)	
Status	Heard of genetic testing	359 (79.8)	<0.001^*^
	Never heard of genetic testing	91 (20.2)	

* *Significant value*.

### Perception of Genetic Testing for Hereditary Disorders

Table 5 shows that majority of the respondents (78.7%) agreed that genetic testing is important. Majority of them agreed that genetic testing is mainly for preventive care purposes (58.7%) and that it should be offered to all newborn babies (71.0%) and pregnant women (68.8%). They also agreed that it is necessary to increase awareness of genetic testing (75.5%) and that the lack of education and knowledge leads to ethical issues concerning the practice of genetic testing (61.0%). Hence, many respondents (81.4%) agreed that laws and policies should be implemented by the government to address the ethical issues concerning the use of genetic testing (Table 5).

**Table 5:** Total and percentage of the respondents' answers pertaining to perception of genetic testing.

**Statements**	**Total (%)**				
	**Strongly disagree**	**Disagree**	**Neutral**	**Agree**	**Strongly agree**
Genetic testing is important.	3 (0.7)	16 (3.5)	77 (17.1)	224 (49.8)	130 (28.9)
Genetic testing is mainly for preventive care purposes.	12 (2.7)	51 (11.4)	121 (27.2)	178 (39.9)	84 (18.8)
Genetic test should be offered to all newborn babies.	7 (1.6)	19 (4.3)	103 (23.1)	219 (49.0)	98 (22.0)
Genetic test should be offered to all pregnant women.	7 (1.6)	25 (5.6)	107 (24.0)	208 (46.6)	99 (22.2)
Knowledge of the genetic background of a disease will help people to live longer.	4 (0.9)	38 (8.5)	118 (26.5)	196 (43.9)	90 (20.2)
Genetic testing does more good than harm.	9 (2.0)	24 (5.4)	112 (25.1)	217 (48.7)	84 (18.8)
Genetic testing will not influence one's health.	9 (2.0)	31 (7.0)	143 (32.0)	188 (41.2)	75 (16.8)
Genetic tests aid in improving one's quality of life.	3 (0.7)	29 (6.5)	115 (25.8)	220 (49.3)	79 (17.7)
Genetic testing tampers with nature.	41 (9.2)	109 (24.4)	184 (41.3)	95 (21.3)	17 (3.8)
Genetic testing opposes religion and their beliefs.	69 (15.4)	129 (28.9)	168 (37.7)	57 (12.8)	23 (5.2)
Lack of education and knowledge of genetics and genetic tests are what raised ethical issues in genetic testing.	8 (1.8)	29 (6.5)	137 (30.7)	218 (48.9)	54 (12.1)
It is necessary to raise awareness of genetic testing.	7 (1.6)	19 (4.3)	83 (18.6)	238 (53.3)	99 (22.2)
Implementation of government laws and policies is needed to ensure the safe and effective use of genetic testing.	7 (1.6)	14 (3.1)	62 (13.9)	208 (46.6)	155 (34.8)

However, irrespective of the positive responses, many respondents remained neutral when asked whether genetic testing tampers with nature (41.3%) and opposes religion and their beliefs (37.7%). Many respondents disagreed (44.3%) with the statements compared with those who agreed (Table 5).

In general, as shown in Table 6, the respondents showed a relatively good perception of genetic testing, which can be associated with their differences in age (*P* = 0.016), ethnicity (*P* = 0.031), educational level, field of study, and whether they have heard of genetic testing or not, with each having a *P*-value of <0.001 (Table 6).

**Table 6:** *P*-value for the respective variables pertaining to questions on perception of genetic testing.

	**Variables**	**Total**	***P*-value**
		***N* (%)**	
Gender	Female	235 (52.2)	0.239
	Male	215 (47.8)	
Age	18–26	215 (47.8)	0.016^*^
	27–34	96 (21.3)	
	35–42	95 (21.1)	
	43–50	44 (9.8)	
Race	Malay	131 (29.1)	0.031^*^
	Chinese	221 (49.1)	
	Indian	89 (19.8)	
	Others	9 (2.0)	
Marital status	Single	286 (63.6)	0.215
	Married	163 (36.2)	
	Divorce	1 (0.2)	
Level of education	A-levels or equivalent	47 (10.4)	<0.001^*^
	Diploma	74 (16.5)	
	Bachelor's	253 (56.3)	
	Master's	29 (6.4)	
	Doctorate	14 (3.1)	
	Others	33 (7.3)	
Field of study	Science	169 (37.6)	<0.001^*^
	Arts	151 (33.6)	
	Others	96 (21.3)	
	No formal	34 (7.5)	
Status	Heard of genetic testing	359 (79.8)	<0.001^*^
	Never heard of genetic testing	91 (20.2)	

* *Significant value*.

## Discussion

### Knowledge of Genetic Testing for Hereditary Disorders

This study shows that many of the respondents have adequate knowledge of genetic testing. This finding can be attributed to the increased reporting of genetic testing in recent years in Malaysia (Yoon et al., 2011; Balasopoulou et al., 2017), which resulted in greater public familiarity of the topic of genetic testing for hereditary disorders. Similar results were observed in some countries in the Middle East, where majority of respondents in Jordan had heard of genetic testing (Mohammad Bagher et al., 2019), while most northern Iranians were interested in using genetic counseling services and genetic tests before marriage (Altaany et al., 2019).

Most of the respondents were unaware that genetic testing can be used to test for various types of cancer. In fact, cancer, particularly breast, and colorectal cancers, can be detected using hereditary cancer or predictive test (Garber and Offit, 2005; Jasperson et al., 2010).

However, good knowledge or public familiarity does not necessarily relate to true understanding, as reported in a study conducted in the United States that explored public understanding of basic genetic concepts (Lanie et al., 2004). Although the respondents can accurately describe certain scientific concepts, it may not be possible for them to translate their basic perceived knowledge of genetic testing during decision making. As reported in a previous survey, more knowledge does not automatically lead to a more positive attitude as more critical and skeptical reactions are often observed among those with highest level of knowledge (Jallinoja and Aro, 2000).

### Awareness of Genetic Testing for Hereditary Disorders

Nearly half of the respondents (50.2%) showed willingness to undergo genetic testing, whereas 24.5% of the respondents gave a negative response and 25.3% of the respondents gave a neutral response. This finding was supported by a similar study in the U.S. population where a large portion (43%) of the general public lacks awareness toward genetic testing (Krakow et al., 2017). In addition, Hann et al. (2017) and Cheng et al. (2016), reported the lack of genetic knowledge as one of the obstacles for patients to participate in gene testing (Cheng et al., 2016; Hann et al., 2017). Cheng et al. also highlighted that the relatively high cost for testing and concerns about discrimination were affecting participants' willingness in uptaking genetic testing. This was in accordance with an earlier study in North Carolina wherein most respondents were concerned about a person's ability to obtain health insurance after receiving genetic tests (Haga et al., 2013). However, these factors were not discussed in this study. We believe that clinical management of patients can be further improved with the aid of healthcare providers or genetic counselors to ascertain the patients' understanding of and beliefs about genetic testing and to increase their willingness to undergo genetic testing (Campbell et al., 2001). In this manner, patients can be better equipped to correct any misconception that they have about genetic testing, thus enabling them to make more informed decisions regarding their health.

Moreover, there is strong support from the respondents that genetic testing should be performed exclusively in hospitals with a doctor's prescription. Furthermore, the majority of the respondents were against the idea of genetic testing kits being sold in stores or through the Internet. Although no similar studies in Malaysia were found, these results were in line with those reported in a survey conducted among the Dutch and Belgium public (Vermeulen et al., 2014; Chokoshvili et al., 2017). Furthermore, in line with some previous studies, majority of the respondents agreed that the public's view and awareness of genetic testing is important (Hann et al., 2017; Krakow et al., 2017).

### Perception of Genetic Testing for Hereditary Disorders

More than half of the respondents recognized the importance of genetic testing and its use for preventive care purposes (Table 3). They also agreed that genetic testing should be offered to all pregnant women and newborn babies. This finding can be attributed to the fact that many of the respondents have a background in science; thus, they were more aware that pregnant women and newborn babies are highly susceptible to or at risk for inherited disorders. A similar study of university students from International Islamic University Malaysia (IIUM) also reported that the majority of the respondents agreed that pregnant women with a family history of inherited disorders should be encouraged to undergo prenatal genetic screening (Sulaiman and Zainuddin, 2018). In addition, our findings are also in line with a recent study by Abdo et al., which reported the receptivity of Jordanian women to non-invasive prenatal genetic screening (Abdo et al., 2018).

Nonetheless, most of the respondents remained neutral when asked whether genetic testing tampers with nature and opposes religion and their beliefs. A minority of respondents agreed to these statements, and this can be related to their ethnicity. A significant difference between ethnicity and perception of genetic testing can be observed. As reported in a previous study in New York city, ethnic and racial identities are associated with the perception of the benefits of and barriers to the application of genetic testing (Sussner et al., 2011). In addition, a study conducted in Jordan also revealed that 12% of the respondents believed that genetic tests were forbidden (Altaany et al., 2019).

Nevertheless, with the high recognition of the importance of genetic testing, the majority of the respondents believe that it is necessary to increase the awareness of this topic as they feel that the lack of education and knowledge of genetics and tests available leads to ethical issues concerning the practice of genetic testing (Table 5). Thus, many respondents agreed that laws and policies should be implemented by the government to ensure the safe and efficient use of genetic testing. The implementation of such policies should be in line with consumers' benefits, such as their eligibility for medical and insurance coverage (Chong et al., 2018). Moreover, a recent study highlighted that the lack of knowledge of genetic testing among healthcare providers was the most significant difficulty faced in California and Malaysia (Amini et al., 2015; Qian et al., 2019). A similar trend was also observed in the Netherlands (Baars et al., 2005) and Italy (Marzuillo et al., 2013). In addition, in accordance with this study, Qian et al. reported in 2019 that expanding newborn screening can be the most beneficial tool to diagnose genetic conditions. However, the authors are agreeable with an earlier study supporting that the public must be treated as a complex body, which is not only composed of scientists, to interpret messages about genetic testing (Bates, 2005). Both scientific understanding and public understanding should be balanced to avoid facing major challenges in promoting the advantages of genetic testing.

### Strengths and Limitations

In general, in terms of the scope of this research, only Malaysians resided in the Klang Valley were included in the survey. Therefore, the findings obtained may not be considered a direct representation of the population of Malaysia as a whole, although the residents of the Klang Valley comprised people from all states of Malaysia. Despite achieving a full response rate of 100%, inaccuracies and biases may be detected because of the choice of location and the respondents chosen. The respondents chosen are those within the age range of 18–50 years old and considered to have a higher capability to understand the topic of the study. Correspondingly, a lower response is obtained from those aged 43 years and above because of language barriers as the questionnaire was developed only in the English language. However, despite the limitations regarding the choice of respondents, this survey was conducted in a large scale, i.e., accounting for the entire Klang Valley, compared with other almost similar research that was conducted in a small scale. Furthermore, this survey provides a first update of Malaysians' knowledge, awareness, and perception of genetic testing for hereditary disorders. Thus, this study is set as the foundation for further similar studies that may be conducted in a larger scale to obtain a better assessment and a more accurate scenario of the knowledge, awareness, and perception of genetic testing of Malaysians.

## Conclusion

The practice of genetic screening for genetic or hereditary disorders is becoming more acceptable worldwide, likewise in Malaysia. In spite of the majority of the residents of the Klang Valley having adequate knowledge and relatively good and positive awareness and perception of genetic testing, there is still a minority of respondents with negative awareness and perception. Thus, the public's knowledge, awareness, and perception of genetic testing should be improved to decrease any stigma or taboo that one may have. For this, media campaigns and government and non-government programs, such as seminars, should be arranged to further educate the public on the beneficial health consequences of genetic testing. Furthermore, the government should implement laws and policies to ensure the safe and efficient use of genetic testing.

## Data Availability Statement

The datasets generated for this study are available on request to the corresponding author.

## Ethics Statement

This study was approved by the SEGi Ethics Committee, with project number SEGi/RIMC/FOP/26/2018. Before the survey, informed written consent was obtained from each respondent, and they were informed that the research was voluntary, confidential and purely for academic purposes.

## Author Contributions

J-JC and H-WT conceived, designed the study, and drafted the manuscript. J-JC acquired and analyzed the data. H-WT critically revised and finalized the manuscript. All authors approved the final version of the manuscript. All authors contributed to the article and approved the submitted version.

## Conflict of Interest

The authors declare that the research was conducted in the absence of any commercial or financial relationships that could be construed as a potential conflict of interest.
